# *Zeylanidiummanasiae*, a new species of Podostemaceae based on molecular and morphological data from Kerala, India

**DOI:** 10.3897/phytokeys.124.33453

**Published:** 2019-06-10

**Authors:** Remya Krishnan, Priyanka Khanduri, Rajesh Tandon

**Affiliations:** 1 University of Delhi, Department of Botany, Delhi-110007, India University of Delhi Delhi India; 2 University of Calcutta, Vidyasagar Metropolitan College, Department of Botany, Kolkata-700006, India University of Calcutta Kolkata India

**Keywords:** Internal transcribed spacer, Malpighiales, Podostemoideae, rheophyte, taxonomy

## Abstract

We present the description of *Zeylanidiummanasiae* (Podostemaceae), a new species from Kerala, India, which is proposed based on molecular, macro- and micromorphological data. This species is characterised by its ribbon-like dichotomous thallus, floriferous shoots produced along the margins and dichotomy of the thallus, inflorescence with two bracts, unequal stigmatic lobes, ellipsoid fruits and large seeds.

## Introduction

Podostemaceae represents a very distinct family of fresh water aquatic angiosperms, with unique evolutionary, ecological, morphological, developmental and embryological attributes (Cook and Rutishauser 2007, Katayama et al. 2016, Khanduri et al. 2014). It is the most diverse family of fresh water aquatic flowering plants, comprising ca. 54 genera and ca. 300 species (Koi et al. 2012, Cheek et al. 2017) distributed worldwide, but with most species presenting restricted distribution and a high degree of endemism (Philbrick et al. 2010). Podostemaceae is subdivided into three monophyletic subfamilies: Podostemoideae, Tristichoideae and Weddellinoideae (monogeneric and monospecific) (Koi et al. 2012). Southern Asia is one of the main centres of diversity for the podostemads, accounting for 17 genera and 80 species from the region (Kato 2016). India harbours 28 species of Podostemaceae, in which 23 are endemics (Khanduri et al. 2014).

The genus, *Zeylanidium* (Tul.) Engl. (subfamily Podostemoideae), is characterised by plants with crustose or ribbon-shaped thalli and caducous leaves. The flowering shoots in these species may be located either in the sinuses of the thallus lobes or borne randomly on the dorsal surface of the thallus (Mathew and Satheesh 1997). Each shoot bears a solitary, terminal and bracteate flower. The other key features include persistent spathella, anisolobous ovarian locules and many seeded capsules (Kato and Koi 2018).

*Zeylanidium* is currently represented by seven species: *Z.olivaceum* Engl., *Z.johnsonii* Engl., *Z.lichenoides* Engl., *Z.maheshwarii* C.J.Mathew & V.K.Satheesh, *Z.sessile* (Willis) C.D.K.Cook & Rutish, *Z.crustaceum* M.Kato and the recently described *Z.tailichenoide* M.Kato & Koi (Mathew and Satheesh 1996, Cook and Rutishauser 2001, Kato et al. 2015, Kato and Koi 2018). Out of these seven species, the status of *Z.johnsonii* Engl. is doubtful as it has never been reported by any study after Engler’s (1930) description. All species are confined to peninsular India, Sri Lanka, Myanmar and Thailand (Mathew and Satheesh 1997, Kato et al. 2015, Kato and Koi 2018). The taxonomic delimitation of the genus is still under dispute. Two species, originally described under the New World genus *Podostemum*, were transferred to *Zeylanidium* by Cusset (1992) as *Z.barberi* (Willis) C. Cusset and *Z.subulatum* (Gardn.) C. Cusset. However, a recent combined morphological and molecular phylogenetic analysis does not support these new combinations (Khanduri et al. 2014). Therefore, these species are here excluded from *Zeylanidium* and it is our opinion that their generic placement requires further studies (pers. observ.).

During field studies in the riverine areas of Kerala, India, between 2014 and 2016, a new morphological variant of *Zeylanidium* was found. The ribbon-shaped specimens with solitary flowering shoots appeared morphologically similar to *Z.lichenoides*, but the fruits were remarkably distinct in size and shape. Detailed morphological and molecular studies revealed that the specimens were different from the remaining species on several other characters. Hence, the specimens are documented and described here as a new species, *Zeylanidiummanasiae*. A detailed description with photographic documentation, illustrations, phylogenetic placement within Podostemaceae and an identification key are provided.

## Material and methods

### Morphology

**Figure 1.**
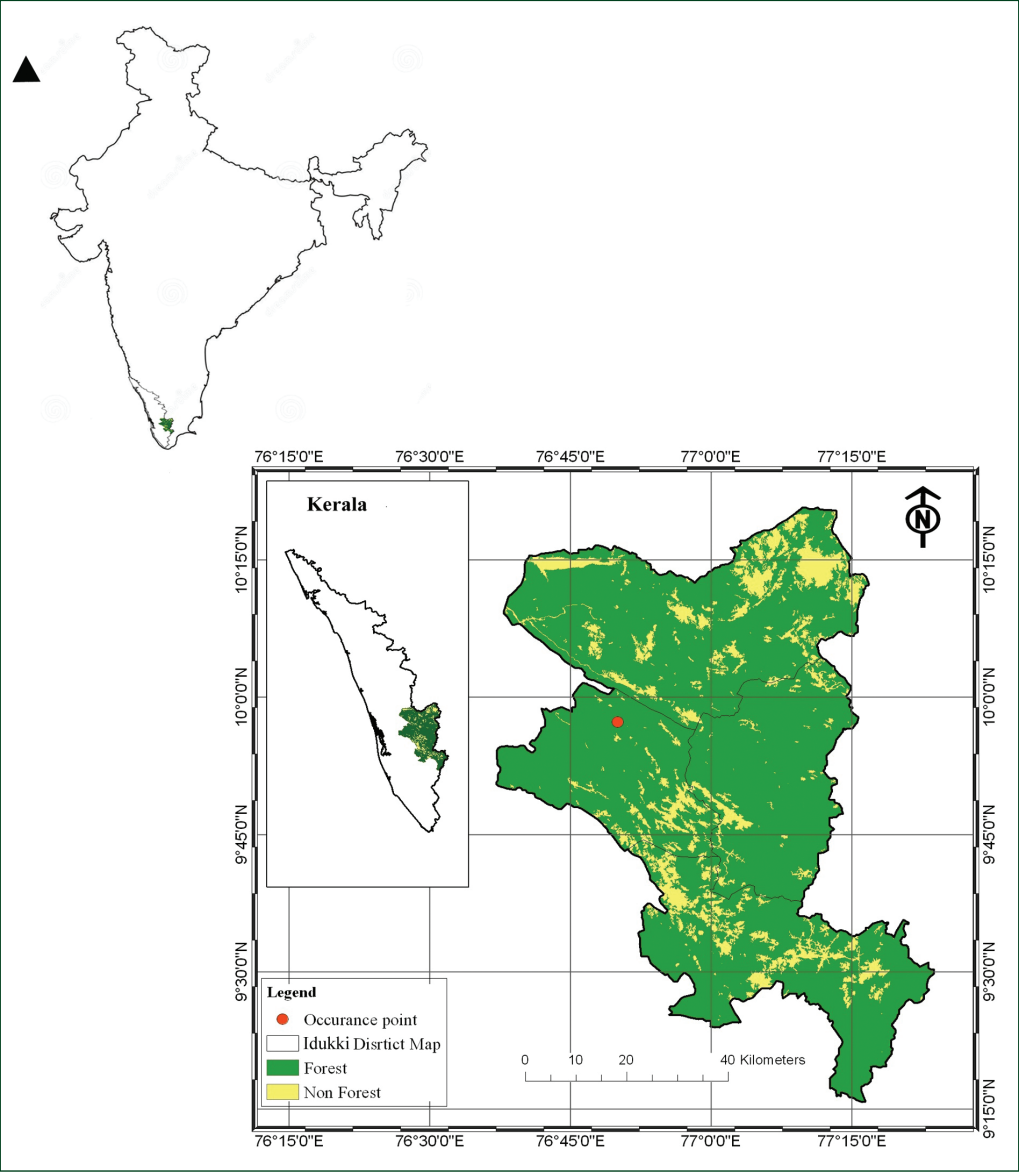
Distribution map of *Zeylandiummanasiae* (red dot). Thommenkuthu Waterfalls, Idukki, Kerala (Map constructed using ArcGIS 9.2 software).

Plant specimens were collected from Thommenkuthu waterfalls, Thodupuzha, Idukki, Kerala, India (9°57'21.59"N 76°50'01.87"E, Fig. 1). Collections were made from various spots at the rapids for three consecutive years (2014–2016). Voucher specimens have been deposited at the Delhi University Herbarium (DUH), Department of Botany, University of Delhi and Calicut University Herbarium (CALI). The morphological details of the plants were recorded in the field and documented photographically. Morphometric details of randomly collected plants (N=30) were measured by using a digital Vernier calliper and calibrated ocular micrometre. The specimens were compared with other members of *Zeylanidium* either by using preserved materials from our spirit collections [i.e. *Z.olivaceum*, *Z.lichenoides*, *Z.sessile* and *Z.maheshwarii* (Suppl. material 1)] or information available in literature (i.e. *Z.johnsonii*, *Z.crustaceum* and *Z.tailichenoides*; Mathew and Satheesh 1997, Suzuki et al. 2002, Kato et al. 2015, Kato and Koi 2018). The differences between the taxa were compiled and are presented below (Table 1, Suppl. material 2). The terminology for vegetative and floral characters follows Mathew and Satheesh (1997), Marinho et al. (2014) and Jäger-Zürn (2003). The distribution map was constructed using ArcGIS 9.2 version (Zhan and Huang 2004).

**Table 1.** Morphometric differences between *Zeylanidiumlichenoides* and *Z.manasiae*.

**Characters**	*** Z. manasiae ***	*** Z. lichenoides ***
Flower length excluding pedicel (mm)	2.38 ± 0.47	1.84 ± 0.10
Anther length (mm)	0.50 ± 0.06 × 0.37 ± 0.05	0.4 ± 0.16 × 0.47 ± 0.02
Pollen production per flower	4273 ± 941	4363 ± 92
Pollen size		
Polar diameter of the dyad (µm)	30.25 ± 2.41	32.08 ± 1.42
Equatorial diameter of the dyad (µm)	19.62 ± 1.99	21.01 ± 1.54
Shape of the pollen	Sub-prolate	Sub-prolate
Ovule production per flower	78 ± 13.70	59 ± 9.27
Pollen: Ovule ratio	55:1	74:1
Capsule size (mm)	1.96 ± 0.25 × 0.84 ± 0.10	1.2 ± 0.20 × 0.8 ± 0.13
Seed number per fruit	60.1 ± 15.63	49 ± 15
Seed size (µm)	248.75 ± 12.70 × 136 ± 8.90	207 ± 1.70 × 108 ± 1.20
Ovule:seed ratio	1.30	1.20

### Anatomy

For anatomical details, flower buds of desired specimens were fixed in Karnowsky’s fixative (Karnowsky 1965) and then processed to prepare resin blocks for sectioning (Feder and O’Brien 1968). Semi-thin sections (4 and 5 μm) were obtained with the help of a rotary microtome, stained with 0.1% toluidine blue O’ (pH 4.4) and mounted in DPX (O’Brien and McCully 1981). The observations were recorded with the help of a photo microscope (Carl Zeiss, Axio scope A1) with an attached digital camera (Axiocam).

### Scanning electron microscopy

For palynological and seed micromorphological studies, anthers and seeds, respectively, were fixed in Karnowsky’s fixative, dehydrated in a graded series of cold acetone (10–100%, 30 min interval each), critical point dried (CPD), mounted on aluminium stubs and coated with gold-palladium alloy before making observations. The samples were examined by using a scanning electron microscope (SEM, JEOL, JSM-6610LV) at the Department of Botany, University of Delhi, India.

### DNA extraction, amplification and sequencing

Genomic DNA was extracted using DNeasy plant mini kit (Qiagen, Amsterdam, Netherlands). DNA amplification and sequencing of the entire ITS region (ITS1, 5.8S and ITS2) were performed using the primers ITS 1 and ITS 2 (White et al. 1990). The polymerase chain reaction (PCR) was executed using standard protocol with one unit of *itaq* (Taq Intron, Intron Biotechnology Inc.), 2.5 μl of 10 X buffer, 2.5 μl dNTPs, 1 μl of 10 pM solution of each primer, 1 μl of genomic DNA and 16.7 μl distilled water. PCR products were purified using QIAquick Gel Extraction Kit (QIAGEN) and the purified product was ligated into a pGEM-T vector (Promega, USA). The ligated mix was transformed using competent *E.coli* DH5α strain. The blue-white selection method was employed for transformation (Sambrook and Russell 2001). Three clones per PCR product were sequenced at SciGenome Labs Pvt. Ltd. (Cochin, India). Contigs were assembled using DNA star Laser gene version 5.07 software (Burland 2000). Nucleotide BLAST was performed to estimate sequence similarity by using the acquired nucleotide sequence as the query. The sequences have been submitted in the GenBank (Suppl. material 3).

### Taxon sampling

ITS sequences of *Z.manasiae* and *Z.maheshwarii* were added to a dataset consisting of 39 species of Podostemaceae, produced by Khanduri et al. (2014). *Hypericumperforatum* L. and *Hypericumkouytchense* H.Lév. were included as outgroups based on the results of Ruhfel et al. (2011). The final data matrix comprised of a total of 43 accessions, representing 41 species of Podostemaceae and the two outgroups. Out of the seven species of *Zeylanidium*, five were included in the present phylogenetic analysis.

### Phylogenetic analysis

ITS sequences of all the taxa were aligned using ClustalX ver. 2.0.11 (Thompson et al. 1997) and checked manually using ClustalW (Thompson et al. 1994). Phylogenetic reconstruction was carried out using MrBayes 3.1 (Ronquist and Huelsenbeck 2003) with the best sequence evolution model i.e. JC model under Model Test version 0.1.1 (Guindon and Gascuel 2003). Analyses were run for 1,300,000 generations until stationarity (standard deviation < 0.01). In each run, trees were sampled after every 100 generations with a sample frequency of 10. All the parameters were summarised after excluding 25% of the samples (burn-in fraction), based on the inspection of log-likelihoods of sampled trees. The summary table provides mean and mode with 95% credibility interval. The potential scale reduction factor approached 1.0 for all the parameters. Branch length information was recorded and averaged across all the retained trees and a majority rule consensus tree was computed to obtain the posterior probabilities (PP). Trees were summarised by the sump burn-in command yielding a cladogram showing PP, clade credibility for each split and a phylogram with mean branch lengths. The values between 0.95 and 1.0 were only taken into consideration for Bayesian analysis.

## Results

### Molecular analyses

DNA sequencing of the ITS region of *Z.manasiae* generated a sequence with 907 bp. This sequence aligned in the genus *Zeylanidium*, confirming its generic identity (Fig. 5). Sequence alignment of different species of *Zeylanidium* showed *Z.manasiae* to be significantly different from the other species, thereby confirms its distinction as a new species. The species was well-nested in *Zeylanidium*. The *Zeylanidium* clade was found to be sister to *Polypleurum*, which is in congruence with earlier molecular studies (Koi et al. 2012, Khanduri et al. 2014). Phylogenetic analysis revealed that all the studied species of *Zeylanidium* grouped together with the exception of *Z.sessile*, which is more closely associated with species of *Polypleurum* (Tul.) Warm. Within the major group, there were three subgroups; one comprising *Z.lichenoides* and *Z.maheshwarii* as sister species (0.63 PP) and the other two consisting of *Z.manasiae* and *Z.olivaceum* which is supported by Bayesian posterior probability of 1.00. *Zeylanidiumsessile* resides alone outside the *Zeylanidium* clade (1.00 PP). We feel that analysis with the addition of *Z.johnsonii*, *Z.crustaceum* and *Z.tailichenoides* might resolve the clade further. All the other major clades including *Polypleurum* were well-supported and are consistent with the earlier phylogenetic analysis (Khanduri et al. 2014).

### Taxonomy

#### Zeylanidium manasiae

Taxon classificationPlantaeMalpighialesPodostemaceae

R.Krishnan, P.Khanduri & R.Tandon sp. nov.

urn:lsid:ipni.org:names:60478968-2

Figs 2 , 3 , 4

##### Diagnosis.

It can be distinguished from the closely related *Z.lichenoides* by the position of floriferous shoots along the margins of thallus, two bracts per floriferous shoot, unequal stigmatic lobes, larger fruits, ellipsoidal capsule and larger seeds.

##### Type.

INDIA. Kerala: Idduki district, Thommenkuthu Waterfalls, River Kaliyar, 9°57'21.59"N 76°50'01.87"E, 64 m alt., 31 Dec 2015, *R. Krishnan & P. Khanduri* 8010 (holotype: DUH accession no. 14378!; isotypes: CALI accession no. 7000!, DUH accession no. 14379!)

##### Description.

Herbs rheophtytic, annual. Thallus 3.79 ± 0.44 mm wide, green to yellow, ribbon-shaped, dorsiventrally flattened, dichotomously branched, attached to the substrate by disc-shaped haptera, 1.14 ± 0.92 mm diam. Leaves produced at the margins and sinuses/branch points of the thallus, in pairs, caducous; blades 2.5–6.75 × 0.26 ± 0.03 mm, subulate, flattened, lacking a midrib. Floriferous shoots produced both marginally and at the branch points of the thallus, solitary, horizontally appressed to the thallus, composed of 2 subulate bracts subtending a single flower, successive shoots 3.56 ± 0.87 mm apart; bracts 2.25–8.20 mm long, caducous, with long caducous apices. Spathella 1.98 ± 0.30 mm long, obovoid, membranous, non-vascularised, persistent, enveloping the flower at pre-anthesis, rupturing longitudinally or irregularly at anthesis. Flowers green, bisexual, zygomorphic, achlamydeous, erect; pedicel measuring 8.32 ± 2.32 mm long in a mature flower; tepals 2, one on either side of the andropodium, 0.83 ± 0.21 mm long, filiform; stamens 2, borne on an andropodium, 0.74 ± 0.15 mm long at anthesis, elongating to 3.67 ± 0.74 mm long at post-anthesis, branched approximately ¼ from the apex, each branch measuring 0.13 ± 0.04 mm long at anthesis, elongating to 0.80 ± 0.10 mm long at post-anthesis, anthers 0.50 ± 0.06 × 0.37 ± 0.12 mm, quadrangular, base bilobed, lobes subequal, dehiscence introrsely rimose; 4273 ± 941 pollen dyads per flower, 30.25 ± 2.42 × 19.62 ± 1.99 µm, tricolpate, microechinate; gynoecium bicarpellate, syncarpic, ovary 2.07 ± 0.28 mm long, ellipsoidal, anisolobous, membranous septum separating two unequal locules, ovules 78 ± 14, anatropous, borne on a swollen axile placenta, style absent, stigma bifid, stigmatic lobes unequal, subconical, the longer 0.48 ± 0.06 × 0.10 ± 0.01 mm, the shorter 0.42 ± 0.06 × 0.08 ± 0.01 mm. Capsule dehiscent, loculicidal capsule measuring 1.96 ± 0.25 × 0.84 ± 0.10 mm and pedicel elongates to 15.55 ± 2.21 mm, bivalved, brown, ellipsoidal, longitudinally ridged, ridges 6, 3 on each valve, one valve persistent, the other deciduous. Seeds 60 ± 15.50 per capsule, 248.75 ± 12.70 × 136 ± 8.90 µm, spermoderm reticulate, cells rectangular with wavy striations.

##### Anatomy.

**Figure 2.**
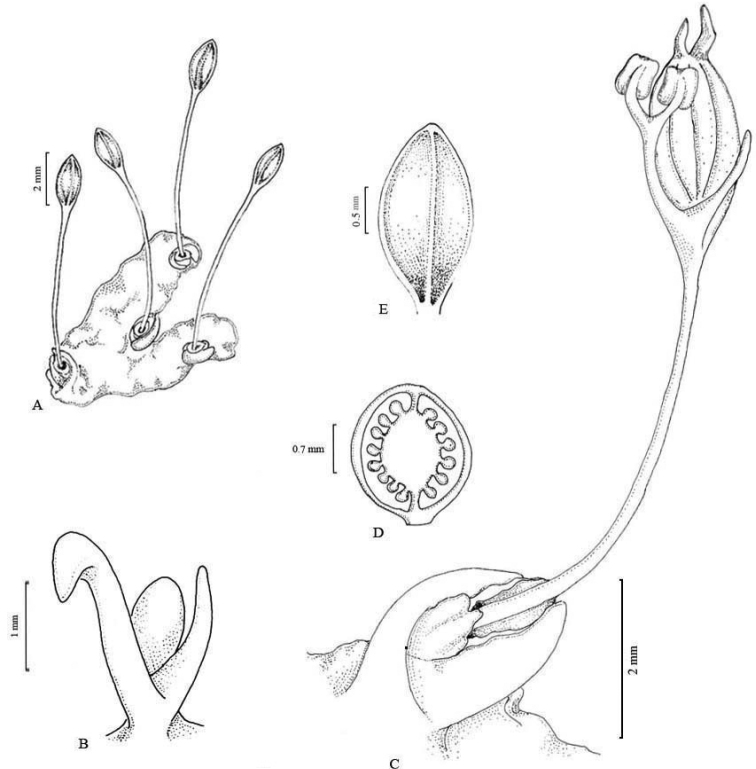
*Zeylandiummanasiae***A** habit showing mature fruits **B** floral bud enclosed in spathella and subtended by two bracts **C** flower showing andropodium, tepals and unequal stigmatic lobes **D** longitudinal section of the ovary, showing unequal locules **E** capsule. Illustration by Rajesh Tandon.

Floral parts of *Z.manasiae* were anatomically investigated. The spathella is non-vascularised and consists of thick-walled polygonal cells. The anthers are of bithecous type, have secretory tapetum and each locule contains pollen dyads. The ovary is pluriovulate and divided into two unequal locules by an apical septum. The ovules are anatropous, bitegmic and tenuinucellate that are borne on a bulbous axile placenta (Fig. 2H).

##### Palynology.

**Figure 3.**
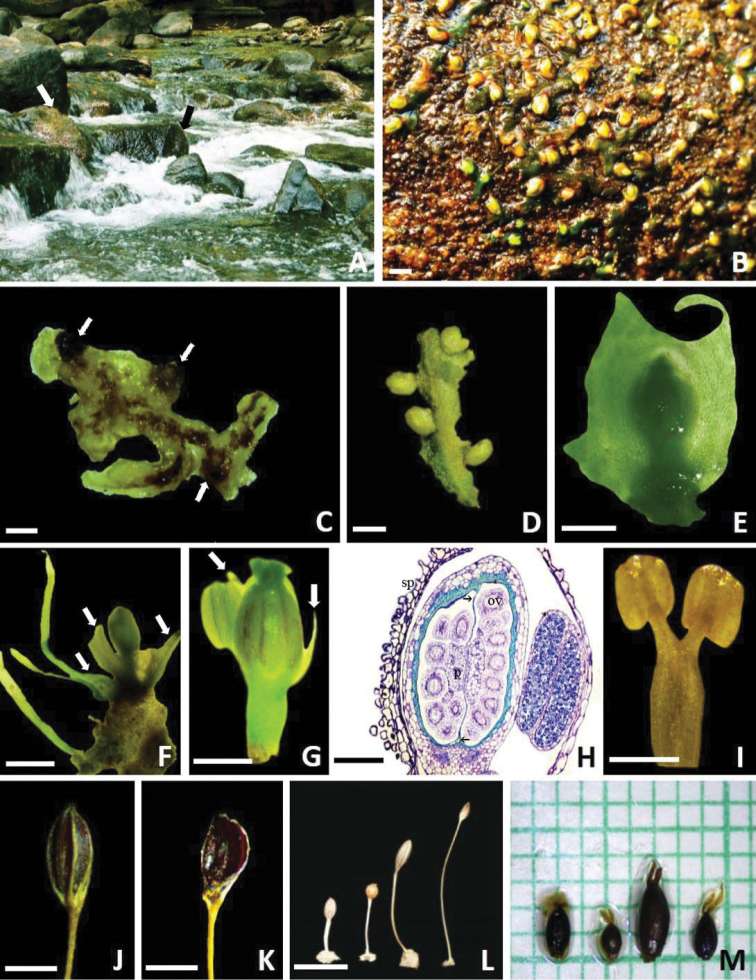
*Zeylandiummanasiae***A** habitat and habit showing plants on exposed rock surface (arrows) **B** habit of the plant showing solitary horizontally appressed flowering shoots **C** ventral surface of the thalli with haptera (arrows) **D** thalli bearing floriferous shoots on margins and point of branching **E** a young flower bud covered by bracts **F** floriferous shoot with flower subtended by two bracts. A pair of leaves can also be seen (arrows) **G** flower with spathella removed showing an anther and two tepals (arrows) **H** longitudinal section of floral bud enclosed in a spathella (sp). The ovary is bilocular and divided into two unequal halves by an apical septum (arrows). Numerous anatropous ovules (ov) are borne on a swollen placenta (p). One of the anthers in section shows a copious amount of dyad pollen **I** forked andropodium with two anthers **J** a mature capsule **K** a dehisced capsule showing persistent valve **L** comparative fruit morphology of congenerics. (Left to right) *Z.maheshwarii*, *Z.lichenoides*, *Z.olivaceum* and *Z.manasiae***M** Comparative morphology of stigma. (Left to right) *Z.maheshwarii*, *Z.lichenoides*, *Z.olivaceum* and *Z.manasiae*. Scale bars: 2 mm (**B**); 3 mm (**C**); 2 mm (**D**); 1 mm (**E**); 2 mm (**F**); 0.5 mm (**G**); 0.2 mm (**H**); 0.5 mm (**I**); 1 mm (**J**); 1 mm (**K**); 5 mm (**L**).

The dyads of *Z.manasiae* are of the acalymmate type and measure 30.25 ± 2.41 µm in length and 19.62 ± 1.99 µm in width. Individual pollen grains are sub-prolate in shape with tricolpate aperture. The exine wall has microechinate ornamentation. The echinations on the apertural surface are larger than those on the non-apertural surface (Fig. 3B).

##### Additional specimens seen (paratypes).

INDIA. Kerala: Idduki district, Thommenkuthu Waterfalls, River Kaliyar, 9°54'00"N 76°46'00"E, 64 m alt., 23 Dec 2016, *R. Krishnan* 8080, (DUH accession no. 14380!). The same locality, 25 Dec 2016, *R. Krishnan* 8081 (DUH accession no.14381!).

##### Etymology.

The specific epithet ‘*manasiae*’ honours the late Dr. Manasi Ram née Ghosh for her contributions to the study of embryology and systematics of Santalaceae (Ghosh 1956) and *Trapa* L. (Lythraceae; Ghosh 1954).

##### Distribution and ecology.

*Zeylanidiummanasiae* is highly endemic and is known from only one location so far, i.e. Thommenkuthu waterfalls (Figs 1 and 3A). According to a previous report, rocks of this waterfall are hornblende biotite gneiss type (Girija 2008). According to Mathew and Satheesh (1996), water in Kaliyar River is well-oxygenated hard water with low chloride content. *Zeylanidiummanasiae* grows along with *Z.lichenoides* and *Z.sessile*. Diatoms (*Cymbella* C.Agardh species) were also observed forming colonies on the spathella of some of the plants.

##### Conservation status.

This species is currently known to occur from a single location in Kerala and, hence, we suggest its placement in the Data Deficient category of IUCN (2017).

##### Phenology.

Flowering and fruiting occurs from December to January when the water level recedes to partly expose the rocks.

## Discussion

**Figure 4.**
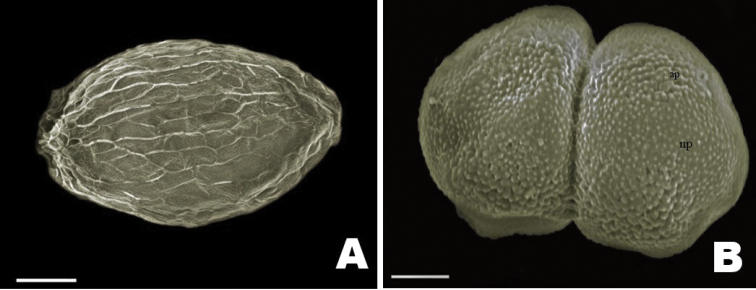
Scanning electron micrographs **A** seed with reticulate spermoderm pattern **B** dyad pollen with micro-echinate ornamentation over the apertural (ap) and non apertural regions (np). Scale bars: 50 μm (**A**); 5 μm (**B**).

*Zeylanidiummanasiae* is a ribbon-shaped, dichotomously branched species, which produces leaves and flowering shoots at the margins and sinuses of the thallus. Based on morphological studies, its closest relative is *Z.lichenoides*, which is also a ribbon-shaped species. However, *Z.manasiae* can be easily distinguished from it on the basis of a number of characters (Table 1): (i) In *Z.manasiae*, leaves and floriferous shoots are present along the margins and sinuses of the thallus (Figs 2A, 3B and D), while in *Z.lichenoides*, they are borne only in the sinuses of the thallus lobes, never along the margins; (ii) There are only 2 bracts per floriferous shoot in *Z.manasiae* (Figs 2C and 3F), while *Z.lichenoides* has 4–6 bracts per floriferous shoot; (iii) *Z.manasiae* has two unequal stigmatic lobes (Figs 2E and 3M), in contrast to *Z.lichenoides*, which has equal lobes; (iv) *Z.manasiae* has comparatively larger fruits and seeds (*Z.manasiae*, capsules 1.96 ± 0.25 × 0.84 ± 0.10 mm, seeds 248.75 ± 12.70 × 136 ± 8.90 µm vs. *Z.lichenoides*, capsules 1.20 ± 0.20 × 0.80 ± 0.13 mm, seeds 207 ± 1.70 × 108 ± 1.20 µm) (Figs 2B, 3J and L; Fig. 4A); and (v) Capsules are ellipsoidal in *Z.manasiae*, whereas they are globose in *Z.lichenoides* (Figs 2B, 3J and L).

*Zeylanidiumtailichenoides* and *Z.sessile*, the other two ribbon-shaped species in the genus, can be easily distinguished from *Z.manasiae* on the basis of (i) unilocular ovary in *Z.tailichenoides* vs. bilocular ovary in *Z.manasiae*; and (ii) sessile flowers and smooth capsules in *Z.sessile* vs. pedunculate flowers and ribbed capsules in *Z.manasiae*. The remaining congenerics (i.e. *Z.olivaceum*, *Z.maheshwarii*, *Z.johnsonii* and *Z.crustaceum*) have crustose thallus with leaves and flowering shoots scattered on the dorsal surface and, hence, are distinct from *Z.manasiae*.

Palynological studies also revealed the presence of tricolpate apertures with micro-echinate exine ornamentation. These characters are similar to the other *Zeylanidium* species, which confirms its generic placement. The structure of the pistil further supports generic identity of the species, since the anisolobous ovary is a characteristic feature of *Zeylanidium*. This characteristic feature separates the genus from *Polypleurum*.

Molecular phylogenetic analysis places *Z.manasiae* in a clade of *Zeylanidium* members which includes *Z.olivaceum*, *Z.maheshwarii* and Z. *lichenoides*. This corroborates the morphological studies done in the present work. The *Zeylanidium* clade is sister to *Polypleurum* within the subfamily of Podostemoideae.

**Figure 5.**
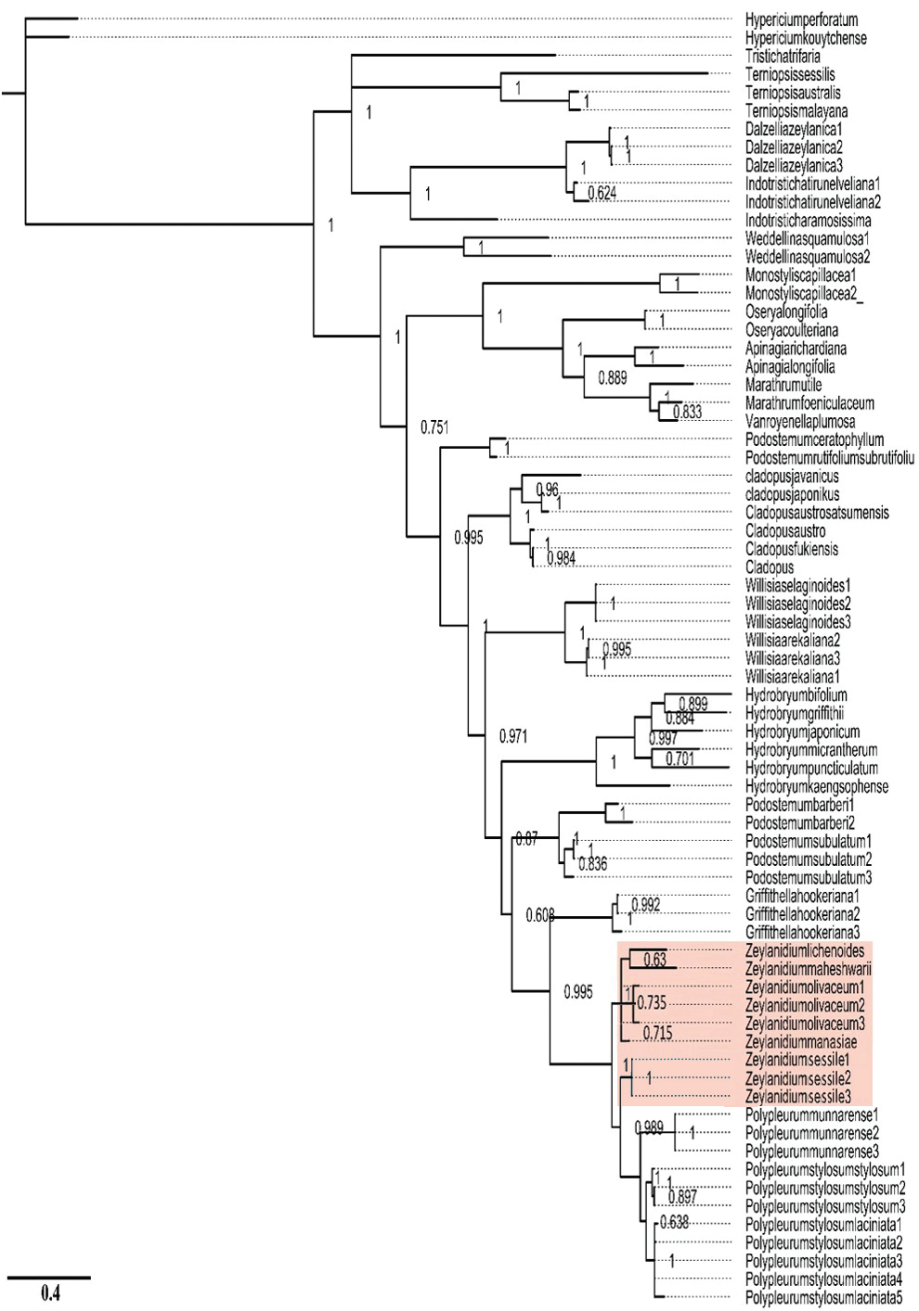
Phylogram of the consensus tree obtained by the Bayesian inference in Mr Bayes. *Zeylanidium* clade has been highlighted. Numbers above the branches indicate values of posterior probabilities.

### Key to the species of *Zeylanidiums.l.*

**Table:** 

1	Thallus ribbon-like; shoots at the sinuses of the thallus or along the margins of the thallus	**2**
–	Thallus crustose; shoots scattered on the dorsal surface of the thallus	**5**
2	Flowers sessile; spathella apex round; capsule smooth	*** Z. sessile ***
–	Flowers pedicellate; spathella apex acute or obtuse; capsules ribbed	**3**
3	Shoots present along the margins and sinuses of the thallus; bracts 2 per floriferous shoot; spathella apex obtuse; stigmatic lobes unequal; capsules ellipsoidal	*** Z. manasiae ***
–	Shoots restricted to the sinuses of thallus; bracts 4–6 per floriferous shoot; spathella apex acute; stigmatic lobes equal; capsules globose	**4**
4	Spathella apex papillate; ovary 2-locular	*** Z. lichenoides ***
–	Spathella apex smooth; ovary 1-locular	*** Z. tailichenoides ***
5	Shoots dimorphic; primary shoot with a tuft of over 20 leaves; secondary shoots with 4–6 leaves	*** Z. olivaceum ***
–	Shoots monomorphic with 4–6 leaves	**6**
6	Leaves up to 10 cm long; bracts 3–4; gynophore absent	*** Z. johnsonii ***
–	Leaves 3–5 mm long; bracts more than 4; gynophore present	**7**
7	Bracts 4–6; stigma bilobed; capsules 8-ribbed	*** Z. crustaceum ***
–	Bracts 6–8; stigma multilobed; capsules 6-ribbed	*** Z. maheshwarii ***

## Conclusion

Based on the evidence drawn from the present work, it is clear that *Z.manasiae* should be recognised as a new species of *Zeylanidium.* The recognition of *Z.manasiae* brings the total number of *Zeylanidium* species to eight. The finding of new species of *Zeylanidium* indicates that the region is splendidly diverse but remains poorly explored.

## Supplementary Material

XML Treatment for Zeylanidium manasiae

## Supplementary materials

Supplementary material 1 Other examined material Data type: species data This dataset is made available under the Open Database License (http://opendatacommons.org/licenses/odbl/1.0/). The Open Database License (ODbL) is a license agreement intended to allow users to freely share, modify, and use this Dataset while maintaining this same freedom for others, provided that the original source and author(s) are credited.Remya Krishnan, Priyanka Khanduri, Rajesh Tandon

Supplementary material 2 Comparative morphology of species of *Zeylanidium**s.l.* Data type: measurement This dataset is made available under the Open Database License (http://opendatacommons.org/licenses/odbl/1.0/). The Open Database License (ODbL) is a license agreement intended to allow users to freely share, modify, and use this Dataset while maintaining this same freedom for others, provided that the original source and author(s) are credited.Remya Krishnan, Priyanka Khanduri, Rajesh Tandon

Supplementary material 3 Source locality, Herbarium Vouchers and GenBank accession numbers of newly incorporated species in the analysis Data type: species data This dataset is made available under the Open Database License (http://opendatacommons.org/licenses/odbl/1.0/). The Open Database License (ODbL) is a license agreement intended to allow users to freely share, modify, and use this Dataset while maintaining this same freedom for others, provided that the original source and author(s) are credited.Remya Krishnan, Priyanka Khanduri, Rajesh Tandon
