# Supplementary material for: Zeylanidiummanasiae, a new species of Podostemaceae based on molecular and morphological data from Kerala, India
Source: PhytoKeys. 2019 Jun 10;124:23–38. doi: 10.3897/phytokeys.124.33453 (PMC6579786; doi:10.3897/phytokeys.124.33453)
Supplement: Supplementary material 1 [file phytokeys-124-023-s001.docx]

**Other Examined materials**

*Zeylanidium olivaceum* Engl*.* INDIA. Kerala: Idukki district, Near Cheeyappara waterfalls, Munnar, 26 Dec 2010, *P. Khanduri & R. Tandon* 102 (DUH accession no. 3107!). *Zeylanidium lichenoides* Engl*.* INDIA. Kerala: Palakkad district, River Bhawanipuzha, 25 Dec 2010 *P. Khanduri & R. Tandon* 105 (DUH accession no. 3105!). *Zeylanidium sessile* (Willis) C.D.K.Cook & Rutish. INDIA. Karnataka: South Canara district, River Killoor 26 Dec 2010 *P. Khanduri & R. Tandon* 105 (DUH accession no. 3114!). *Zeylanidium* *maheshwarii* C.J.Mathew & V.K.Satheesh INDIA. Kerala: Thommenkuthu Waterfalls, River Kaliyar, 31 Dec 2015, *R. Krishnan & P. Khanduri* 3116 (DUH accession no. 14377!)
